# Agreement of pain assessment between nurses and patients in the Emergency Department

**DOI:** 10.1186/cc9877

**Published:** 2011-03-11

**Authors:** M Modanloo, H Abdollahi, N Behnampour

**Affiliations:** 1 Golestan University of Medical Sciences, Gorgan, Iran

## Introduction

Pain is one of the most common reasons why patients visit the Emergency Department (ED) and is a healthcare problem for patients [1,2]. The purpose of this study was to determine the agreement between patient self-reported pain intensity and nurse pain assessment in the ED.

## Methods

A purposive sample of 100 patients and 36 nurses in the triage and clinical area within the ED was selected from 5Azar Hospital in Gorgan. The questionnaire included two components: participant characteristics and the Numeric Rating Scale (NRS). A questionnaire was administered twice to each patient. In triage the patients were asked to rate their pain intensity. Separately, the nurses assessed the patient's pain intensity. This process was repeated with the same patients after referring them to a clinical area within the ED, but the nurses were different. Gathered data were described by frequency distribution tables and analyzed by Wilcoxon, Mann-Whitney and Spearman tests. *P *< 0.05 was considered significant.

## Results

Most of the patients were male (61%), with mean age of 39.16 ± 16.83 years. Fifty-four percent of the patients had chronic pain. Most of them had a diagnosis of abdominal pain and chest pain (61%). In the triage, the mean nurses' pain intensity score was significantly lower than patients' score (7.60 ± 2.1 vs. 9.13 ± 1.26), as significant differences in mean scores were observed (*P *< 0.001). In the clinical area, patients' scores were also significantly higher than nurses 7.36 ± 2.56 and 5.94 ± 2.33, respectively (*P *< 0.001). Nurses significantly underestimated pain on the NRS. See Table 1.

**Table 1:** Patients' (*n *= 100) and nurses' (*n *= 36) pain intensity scores

	Triage area	Clinical area	Total
Nurses	7.60 (2.1)	5.94 (2.33)	6.77 (2.36)
Patients	9.13 (1.26)	7.36 (2.56)	8.24 (2.20)
Correlation	*r *= 0.612	*r *= 0.373	*r *= 0.528

Data presented as mean (SD).

## Conclusions

The findings have implications for the management of patients' pain by highlighting the need for more accurate pain assessment. Further research is required to elucidate the way in which nurses and patients conceptualize pain and to understand better the process of pain assessment in clinical nursing practice.
